# Retinal pathology in the PPCD1 mouse

**DOI:** 10.1371/journal.pone.0185094

**Published:** 2017-10-05

**Authors:** Anna L. Shen, Susan M. Moran, Edward A. Glover, Leandro B. Teixeira, Christopher A. Bradfield

**Affiliations:** 1 The McArdle Laboratory for Cancer Research, Department of Oncology, School of Medicine and Public Health, University of Wisconsin-Madison, Madison, Wisconsin, United States of America; 2 McPherson Eye Research Institute, University of Wisconsin-Madison, Madison, Wisconsin, United States of America; 3 Department of Pathobiological Sciences, School of Veterinary Medicine, University of Wisconsin-Madison, Madison, Wisconsin, United States of America; 4 Wisconsin Institute for Discovery, University of Wisconsin-Madison, Madison, Wisconsin, United States of America; University of Iowa, UNITED STATES

## Abstract

Retinal phenotypes of the PPCD1 mouse, a mouse model of posterior polymorphous corneal dystrophy, have been characterized. PPCD1 mice on the DBA/2J background (D2.*Ppcd1*) have previously been reported to develop an enlarged anterior chamber due to epithelialization and proliferation of the corneal endothelium and subsequent blockage of the iridocorneal angle. Results presented here show that D2.*Ppcd1* mice develop increased intraocular pressure (IOP), with measurements at three months of age revealing significant increases in IOP. Significant retinal ganglion cell layer cell loss is observed at five months of age. D2.*Ppcd1* animals also exhibit marked degeneration of the outer nuclear layer in association with hyperplasia of the retinal pigment epithelium. Evidence of retinal detachment is present as early as three weeks of age. By 3.5 months of age, focal areas of outer nuclear layer loss are observed. Although the *Gpnmb*^*R150X*^ mutation leads to increased IOP and glaucoma in DBA/2J mice, development of anterior segment and retinal defects in D2.*Ppcd1* animals does not depend upon presence of the *Gpnmb*^*R150X*^ mutation.

## Introduction

Human posterior polymorphous corneal dystrophy (PPCD, OMIM #122000) is an autosomal dominant disorder characterized by metaplasia of the corneal endothelium [1–4]. PPCD and autosomal dominant congenital hereditary endothelial corneal dystrophy, as well as the sporadic disorder, iridocorneal endothelial syndrome (ICE), exhibit overlapping clinical and pathological features, including ultrastructural changes and abnormal patterns of cytokeratin expression consistent with epithelialization of the corneal endothelium. A range of clinical consequences has been reported, from minimal visual impairment to development of corneal edema, retrocorneal membranes, corneal opacification, and increased intraocular pressure. Glaucoma has been reported in 15–40% of cases depending on the population studied [5]. Steepening of the cornea has been reported, suggesting a link to keratoconus [6], as well as a single case of colloid drusen formation [7]. Human PPCD has been linked to three chromosomal loci, *PPCD1 (OVOL2)* on Chromosome 20 [5, 8–10], *PPCD2* (*COL8A2*) on Chromosome 8 [11], and *PPCD3* (*ZEB1)* on chromosome 10 [12]. Mutations in the promoter of *OVOL2* have recently been implicated in human PPCD1 [10, 13, 14].

The PPCD1 mouse exhibits many features of human posterior polymorphous corneal dystrophy, including corneal endothelial cell metaplasia and proliferation and inappropriate pan-cytokeratin expression [15]. We have shown that mouse PPCD1 is linked to a 3.9 Mbp chromosomal inversion, flanked by 81 Kbp and 542 bp deletions, and located in a region of mouse Chromosome 2 syntenic to the human *PPCD1* locus [16], referred to as *Ppcd1* (Mouse Genome Informatics, http://www.informatics.jax.org/ [17]). This chromosomal rearrangement truncates the coding sequences of two genes, *Csrp2bp* and *Dzank1*, and results in production of novel truncated and fusion transcripts as well as upregulation of the adjacent gene, *Ovol2*.

Anterior chamber phenotypes of the PPCD1 mouse on the DBA/2J background (D2.129(B6)-*Ppcd1*/J, hereafter referred to as D2.*Ppcd1*) have been described [15, 16]. Briefly, D2.*Ppcd1* eyes exhibit corneal endothelial cell metaplasia with inappropriate pan-cytokeratin staining. Corneal neovascularization and F4/80 positive cells are present. Proliferation of the epithelialized corneal endothelium produces occlusion of the angle leading to a grossly observable enlarged anterior chamber. The globe is also enlarged. Corneal endothelial cell metaplasia begins at approximately postnatal day 3 and an enlarged anterior chamber is apparent upon visual observation as early as postnatal day 14. In this manuscript, we have characterized the retinal phenotypes of the PPCD1 mouse. We demonstrate that D2.*Ppcd1* animals also develop increased intraocular pressure and loss of retinal ganglion cell layer (RGCL) cells, as well as abnormalities of the retinal pigment epithelium leading to outer nuclear layer (ONL) degeneration.

## Materials and methods

### Animals

All procedures conformed to the principles embodied in the ARVO Statement for the Use of Animals in Ophthalmic and Vision Research (www.arvo.org) and the recommendations in the Guide for the Care and Use of Laboratory Animals of the National Institutes of Health. Procedures were also approved by the Animal Care and Use Committee, School of Medicine and Public Health, University of Wisconsin-Madison (Protocol Number M00578). The PPCD1 mouse has been described previously [15, 16] and is maintained on the D2 background. Homozygotes do not survive gestation and all studies were carried out on heterozygotes, designated D2.*Ppcd1*, with wildtype littermates as controls. DBA/2J (D2) and DBA/2J-*Gpnmb*^*+*^/SjJ (JAX 007048, referred to as D2-*Gpnmb*^*+*^) mice were obtained from The Jackson Laboratory, Bar Harbor ME. D2.*Ppcd1* animals were bred to D2-*Gpnmb*^*+*^ for two generations to obtain progeny homozygous for wildtype *Gpnmb*. Animals heterozygous for *Ppcd1* and homozygous for wildtype *Gpnmb* are designated D2.*Ppcd1 Gpnmb*^*+*^. Animals were housed in groups of two to four in plastic cages with corncob bedding, maintained on a 12-hour light:dark cycle, and provided with lab chow (Mouse diet 9F 5020; PMI Nutrition International) and water *ad lib*. Euthanasia was carried out using carbon dioxide exposure and death verified by the absence of both respiration and a heartbeat prior to tissue collection or disposal.

### Genotyping

Tail DNA was isolated using the Puregene reagent according to the manufacturer’s instructions (Gentra, Minneapolis MN) and stored in 10 mM Tris, pH 8.0, 0.1 mM EDTA. Oligonucleotides were obtained from Integrated DNA Technologies (Coralville IA). PCR genotyping for the *Ppcd1* allele was carried out as described in Shen et al [16]. Genotyping for *Gpnmb*^*R150X*^ was carried out by PCR amplification with the oligonucleotides, 5’-TGGGACTGACATCTGACCTG-3’ and 5’-GACAAAGCTCCATTTCTTCCA-3’, followed by digestion of the amplified product with *Pvu2* (New England Biolabs), to identify the presence of the *Gpnmb*^*R150X*^ mutation [18]. PCR reactions contained 10 mM Tris, pH 9, 50 mM KCl, 0.1% Triton X100, 1.25 mM MgCl_2_, 0.1 mM dNTPs, 0.2 μM of each primer, 2.5 units *Taq* polymerase (Promega), and 25 ng tail DNA. Cycling conditions were 94°, 2 min; [94°, 30 sec; 58°, 40 sec; 72°, 1 min] for 40 cycles; 72°, 5 min.

### Intraocular pressure

Mice were anesthesized with isoflurane and intraocular pressure determined using the TonoLab (TioLat, Helsinki, Finland), according to the manufacturer’s instructions. All measurements were done between 1 and 3 PM.

### Histology

For histological characterization, animals were sacrificed by CO_2_ inhalation and enucleated eyes fixed overnight at 4°C in 10% (v/v) formalin in phosphate-buffered saline, pH 7.2, dehydrated in graded ethanol, and processed for routine paraffin embedded light microscopy. Sections were stained with hematoxylin and eosin (H&E). ONL loss was quantified in H&E sections which passed through both the pupil and the optic nerve. ONL thickness was measured at 50 μm intervals beginning at the optic nerve and continuing for 700 μm. Nissl staining of the retinal ganglion cell layer (RGCL) was carried out as described by Li et al. [19], with the exception that retinas were flattened after, rather than before, staining with cresyl violet. RGCL cells were quantified using the Cell Counter feature of Image J to mark and count individual RGCL cells [20]. Cell counts were determined for 4 0.33 mm^2^ fields, one from each quadrant of the retina.

### Transmission electron microscopy

After perfusion fixation with 1.2% paraformaldehyde, 0.8% glutaraldehyde, 0.1 M sodium phosphate, pH 7.2, globes were dissected from the orbit and immersion-fixed in 2% paraformaldehyde (PFA) and 2.5% glutaraldehyde in 0.1 M phosphate-buffered saline (PBS; pH 7.4) at 4°C overnight. Globes were sampled on the dorso-ventral plane and the analysis was focused posterior segment of the globes. Samples were processed for routine transmission electron microscopy. Briefly, fixed globes were treated with 1% OsO_4_ in PBS for 2 hours at room temperature followed by three 10-minute washes with 0.1 M sodium acetate buffer. Tissues were then stained with 2% uranyl acetate in sodium acetate buffer for 1 hour at room temperature, washed in buffer, dehydrated in a graded ethanol series (40%–100%), and infiltrated with propylene oxide-812 resin (1005 Embed 812; EMS, Fort Washington, PA). The samples were embedded with fresh 100% 812 resin in molds and polymerized in a 60°C oven for 36 hours. Ultrathin sections (90 nm) were analyzed using a JEOL 100CX electron microscope (JEOL Ltd., Tokyo, Japan).

### Optic nerve axon quantification

Axons in the optic nerve were quantified using a semi-automated targeted counting method previously described [21]. Briefly, optic nerves were dissected and a cross section of a region 0.5 mm posterior to the globe was processed for epoxy embedding as described above. Semi-thin (1μm) sections were stained with 1% p-phenylenediamine (PPD) and full optic nerve cross sections were analyzed from each optic nerve. Images from the PPD stained optic nerve sections were obtained using a bright light microscope Olympus BX43 (Olympus Inc., Center Valley, PA) with attached Olympus DP72 digital camera (Olympus Inc.), and captured using commercially available image-analysis software (CellSens Dimension^®^, Olympus Inc.). The previously described a semi-automated targeted counting method was applied to the optic nerve sections and total numbers of axon were estimate for each optic nerve.

### Statistical analysis

Statistical analysis was carried out using the software program Mstat (Dr. Norman Drinkwater, University of Wisconsin-Madison, Madison WI, http://www.mcardle.wisc.edu/mstat/) or the statistical functions of Excel. P values were determined using the Kruskal-Wallis test (IOP and RGCL cell and axon counts) or Student’s t test (ONL thickness).

## Results

### Increased intraocular pressure in D2.*Ppcd1* mice

**Fig 1:**
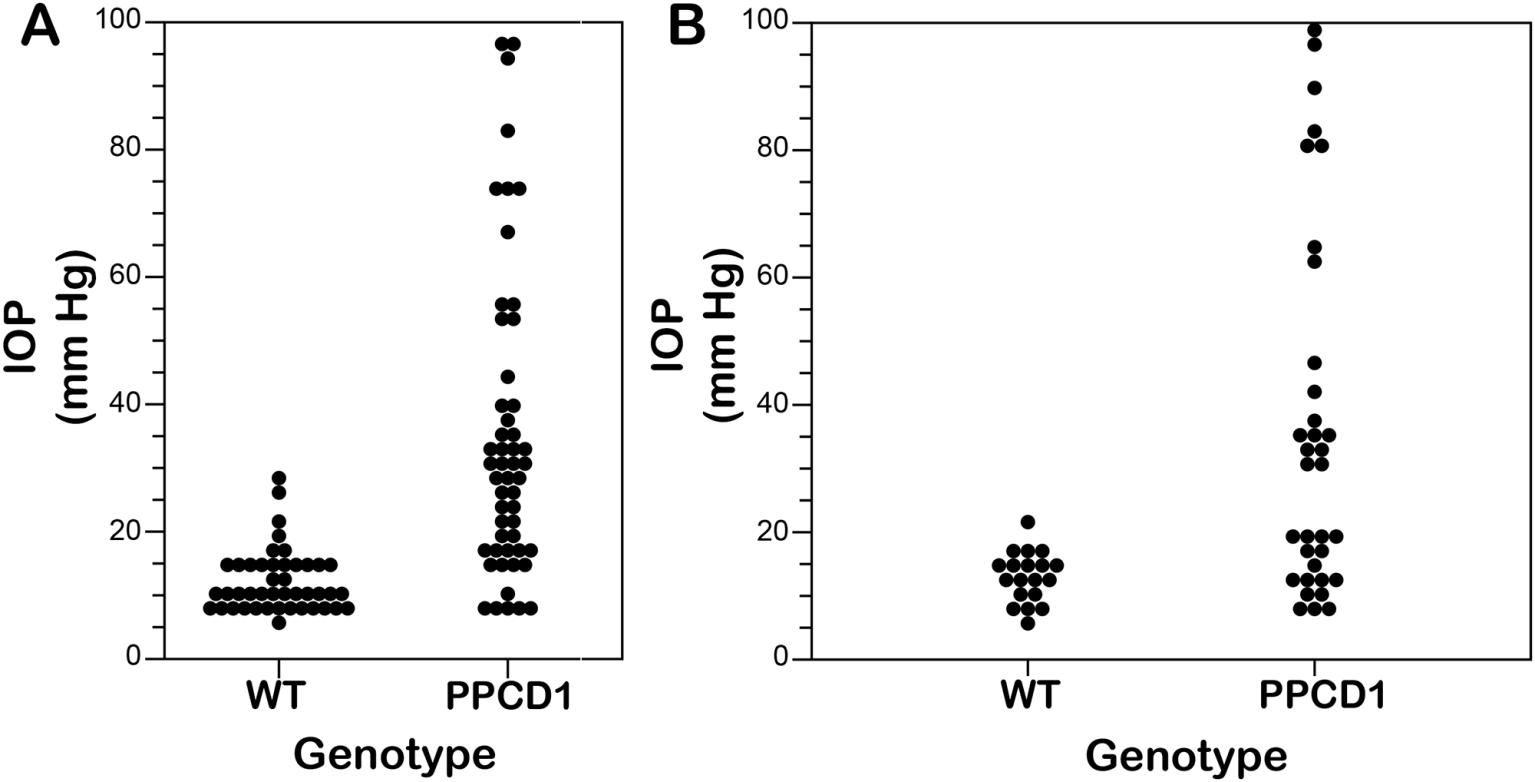
PPCD1 intraocular pressure at 3 months of age. Each point represents a single eye. A. Wildtype (WT) and PPCD1 (PPCD1) animals on the D2 genetic background. B. Wildtype (WT) and PPCD1 (PPCD1) animals on the DBA/2J-*Gpnmb*^*+*^/SjJ genetic background.

Evidence of distension of the anterior chamber is observed at postnatal day 5 [15]. S1 Fig shows marked enlargement of the anterior chamber at postnatal day 8 in D2.*Ppcd1* animals. Tonolab measurements showed a mean intraocular pressure (IOP) in 3 month old wildtype DBA/2J eyes of 12.1 ± 4.4 mm Hg (mean ± SD). Individual values are shown in Fig 1A. IOP in D2.*Ppcd1* eyes is significantly increased, to 35.0 ± 24.2 mm Hg (P < .001), with individual values ranging as high as 97 mm Hg. A large variation in IOP was observed in D2.*Ppcd1* eyes. Variation in IOP and eye size were present in eyes from a single animal. Eyes with normal or smaller than normal globe sizes (S2 Fig) and histologic evidence of phthisis generally had lower pressures; however low IOP values were also observed in animals with enlarged anterior chambers.

### No effect of *Gpnmb*^*R150X*^ on corneal endothelial cell metaplasia and IOP

The DBA/2J strain carries the *Tyrp1*^*b*^ and *Gpnmb*^*R150X*^ mutations, leading to, respectively, iris stromal atrophy and iris pigment dispersion [22]. Homozygosity of *Gpnmb*^*R150X*^ contributes to severe iris disease and elevated IOP, and, through the ages previously studied (15 months), is required for development of glaucoma in aged D2 mice [23]. To investigate the influence of *Gpnmb* on development of the mouse PPCD1 phenotype, D2.*Ppcd1* animals were crossed to D2-*Gpnmb*^*+*^, which is coisogenic with D2 but carries a functional *Gpnmb* allele and does not develop elevated IOP or glaucoma [23]. Anterior chamber pathology in D2.*Ppcd1 Gpnmb*^*+*^ animals is indistinguishable from that of D2.*Ppcd1* animals, with grossly observable enlarged eyes and histologic evidence of corneal endothelial cell metaplasia and an occluded iridocorneal angle (S3 Fig). As with D2.*Ppcd1* animals, IOP values ranged as high as 97 mm Hg (Fig 1B). Mean IOP in D2.*Ppcd1 Gpnmb*^*+*^animals was 36.41 ± 28.5 mm Hg, significantly higher than the IOP of 13.0 ± 4.0 mm Hg seen in wildtype D2-*Gpnmb*^*+*^ littermates (P = .0006).

### Retinal ganglion cell loss in D2.*Ppcd1* animals

**Fig 2:**
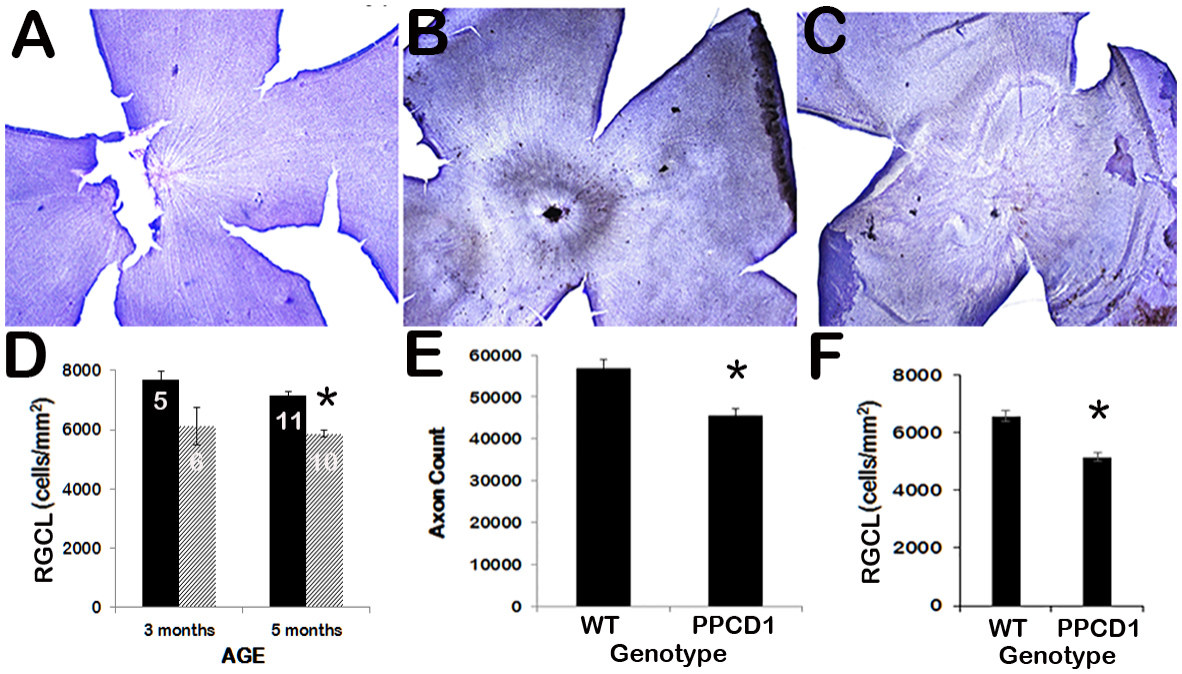
RGCL cell and axon loss in D2.*Ppcd1* eyes. A. Nissl staining of wildtype D2 retina, age 3 months, magnification 40X. B and C. Nissl staining of retinas of D2.*Ppcd1* animals, age 3 months, magnification 40X. D. RGCL cell counts of wildtype (black bars) and PPCD1 (striped bars) animals on the D2 background at 3 and 5 months of age. Values are mean ± SEM. Number of eyes is shown in white. * indicates P = 0.005. E. Axon counts of wildtype (WT) and PPCD1 (PPCD1) animals on the D2 background at 5 months of age. Values are mean ± SEM. * indicates P < 0.005. F. RGCL cell counts of wildtype (WT) and PPCD1 (PPCD1) animals on the D2-*Gpnmb*^*+*^genetic background at 5 months of age. Values are mean ± SEM. * indicates P < 0.005.

Nissl staining of flat-mounted retinas from 5-month-old wildtype D2 eyes showed a regular pattern of cells in the retinal ganglion cell layer (RGCL) radiating out from the optic nerve (Fig 2A). In contrast, D2.*Ppcd1* eyes showed patchy areas of RGCL cell loss as well as a disorganized architecture (Fig 2B and 2C). Mean RGCL cell counts were decreased at 3 months of age, but the decrease did not reach statistical significance (P = 0.12). At 5 months of age, RGCL cell counts in D2.*Ppcd1* eyes were decreased by approximately 20% compared to normal littermates (P = 0.005, Fig 2D). Measurements of axon number correlated with RGCL cell numbers (Fig 2E). Axon counts significantly decreased, to 80% of wildtype (P = 0.004), at 5 months of age. Optic nerve cupping was not observed in D2.*Ppcd1* eyes. RGCL cell counts in D2.*Ppcd1 Gpnmb*^*+*^ animals were also decreased by approximately 20% relative to D2-*Gpnmb*^*+*^ littermates (P = 0.004, Fig 2F) at 5 months of age.

### Retinal detachment in D2.*Ppcd1* eyes

**Fig 3:**
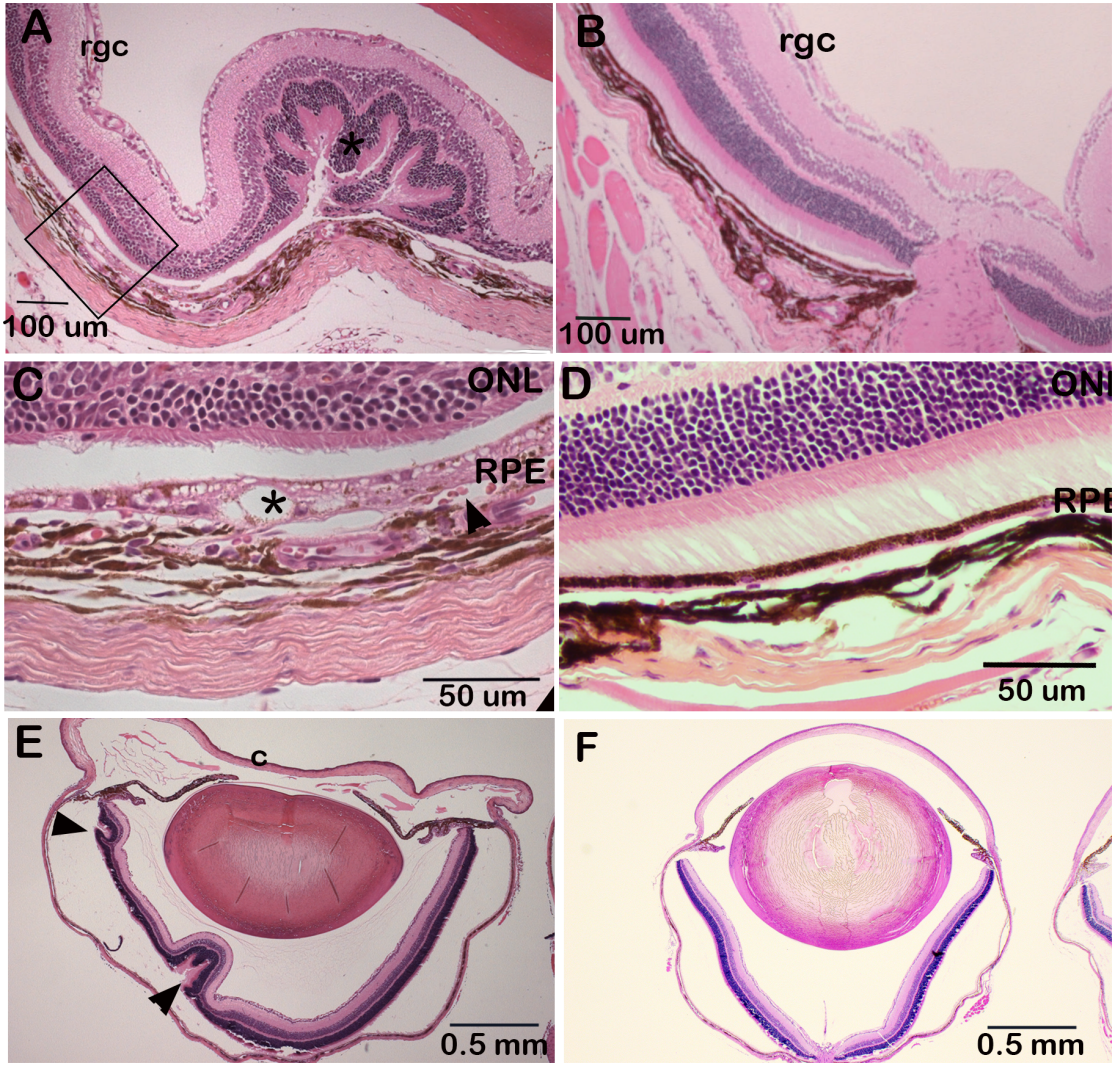
Retinal detachment in D2.*Ppcd1* eyes. H & E—stained sections. A. D2.*Ppcd1* retina, age 3 months. * indicates retinal detachment and fold. Area marked by the rectangle is shown in Panel C at higher magnification. B. Wildtype D2 retina, age three months. C. Area outlined in Panel A. * indicates an intracytoplasmic vacuole and the arrowhead points to a blood vessel. D. Wildtype D2 retina, age three months. E. D2.*Ppcd1* eye, age three weeks. Arrowheads point to retinal folds. F. Wildtype D2 eye, age three weeks.

Histologic examination shows evidence of focal areas of retinal detachment in D2.*Ppcd1* eyes (Fig 3A), accompanied by neovascularization and abnormalities of the retinal pigment epithelium (RPE), including intracytoplasmic vacuoles, multifocal loss of melanin granules (hypopigmentation) and areas of RPE hyperplasia (Fig 3C). Examination of H&E sections of eyes from three-week old animals shows the presence of retinal folds in at least one eye in 3 of 7 of D2.*Ppcd1* animals. (Fig 3E).

### Outer nuclear layer and retinal pigment epithelium abnormalities in D2.*Ppcd1* eyes

**Fig 4:**
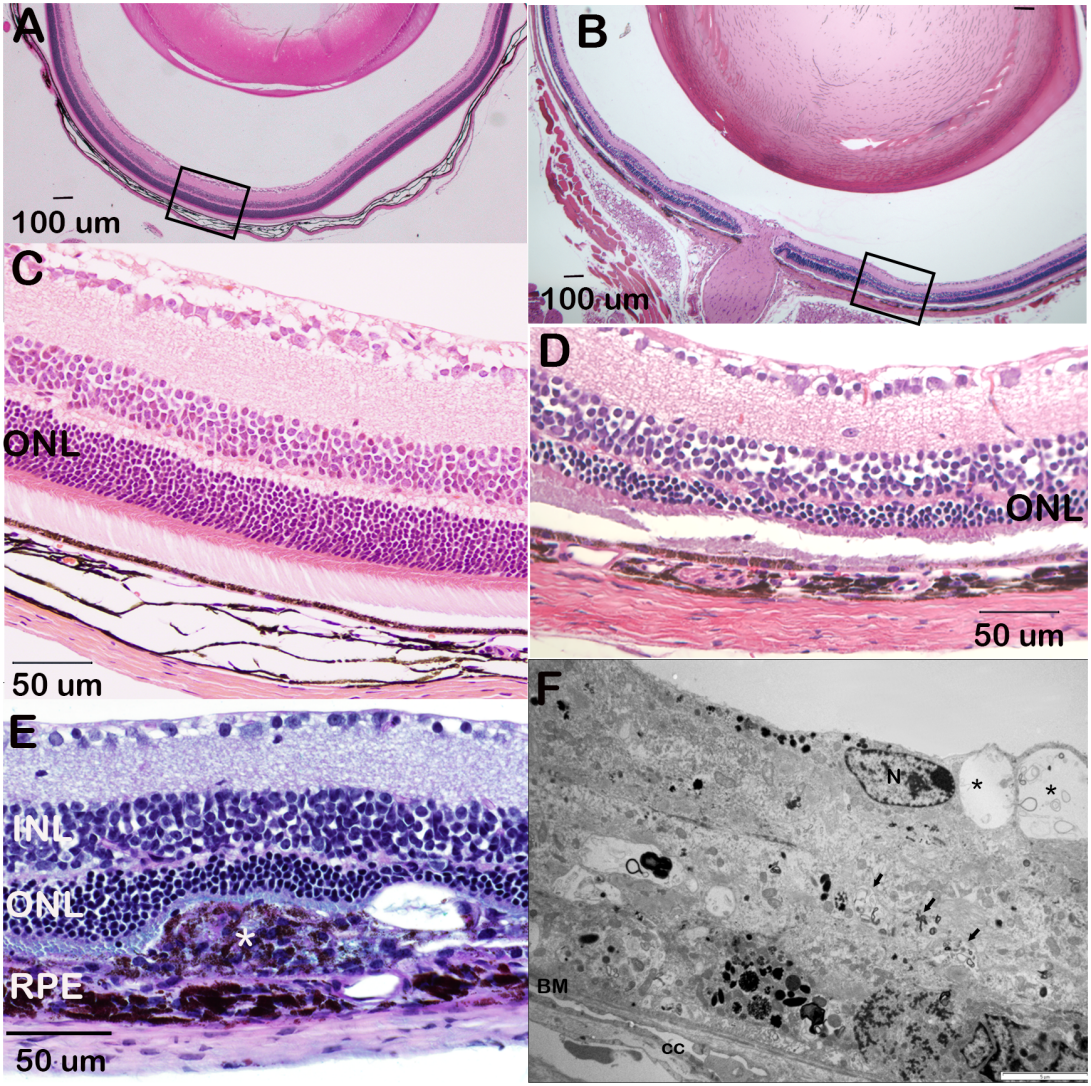
Outer nuclear layer thinning and retinal pigment epithelium hyperplasia in D2.*Ppcd1* eyes. Age, 3.5 months. A. H & E section of a wildtype D2 eye. Outlined area is shown in Panel C. B. H & E section of D2.*Ppcd1* eye showing multifocal thinning of the ONL and a relatively normal optic nerve head. Outlined area is shown in Panel D. C. Higher magnification of area outlined in Panel A. D. Higher magnification of area outlined in Panel B, showing relatively normal RGC layer and thin ONL. E. D2.*Ppcd1* retina, Alcian blue-PAS stain. The * indicates a focal area of RPE hyperplasia compressing the outer retina. F. Ultrastructural appearance of the proliferating RPE cells in D2.*Ppcd1* retina. Note the multiple layers of cells presenting cytoplasmic vacuolization (*) and intracellular accumulation of organelle debris (arrows). Scale bar (white) indicates 1 μm. N: RPE cell nuclei, BM: Bruch’s membrane, CC: choriocapillaris.

**Fig 5:**
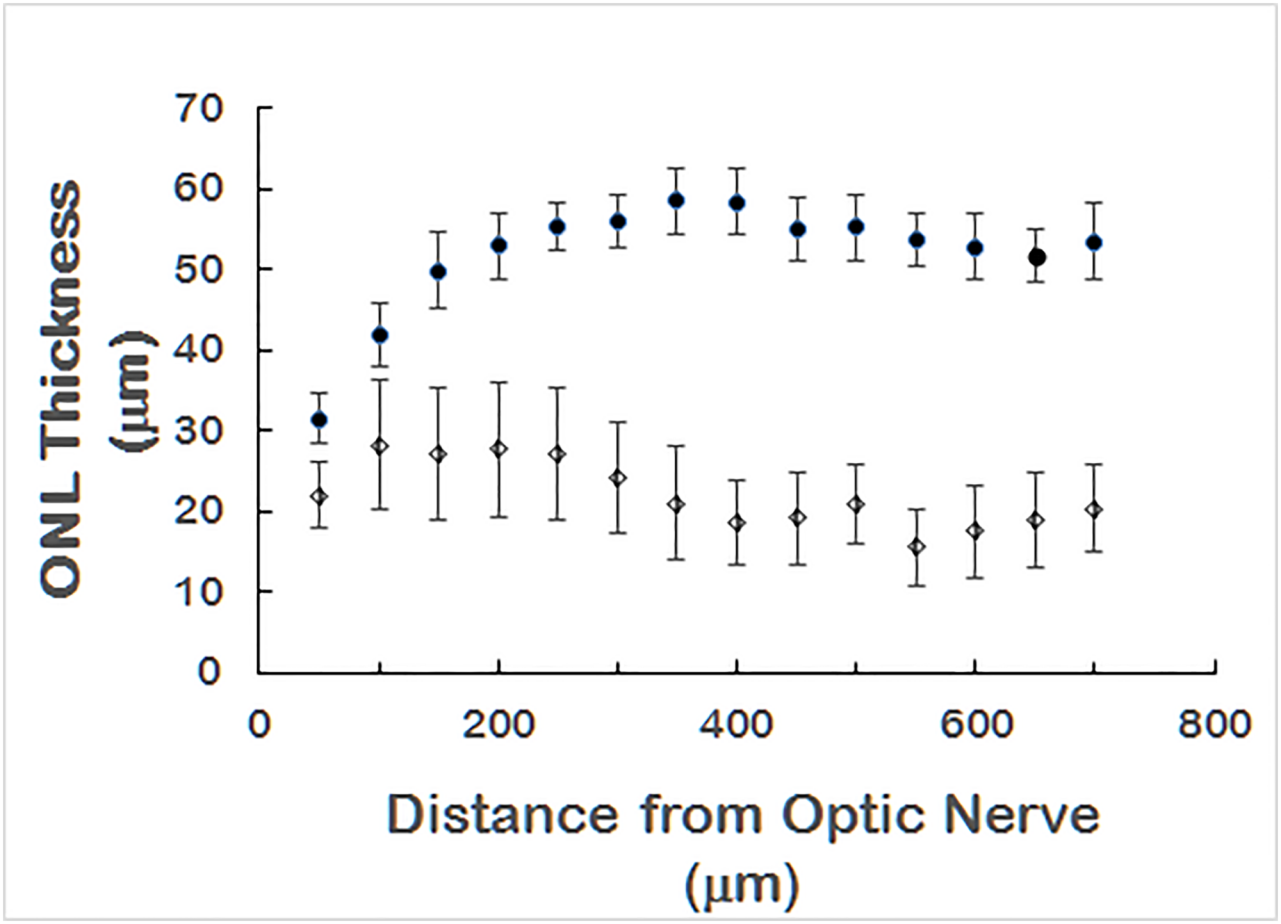
Quantitation of ONL thickness. Age 2.5 to 3.5 months. Values are mean +/- SD. n = 10 eyes for D2.*Ppcd1* (open circles) and 6 eyes for wildtype D2 (closed circles). P < 0.05 for all points except 50 μm.

Loss of the outer nuclear layer was observed in D2.*Ppcd1* eyes. Fig 4B and 4D shows an example of ONL loss in a 3.5-month old animal. Areas of ONL loss did not correlate with areas of RGCL cell loss (Fig 4D and 4E). Examination of eyes of 21 animals, age 2.5 to 3.5 months, showed that 100% of D2.*Ppcd1* animals exhibited at least one area of ONL loss in at least one eye (S4 Fig). Fig 5 shows mean ONL thickness as a function of distance from the optic nerve for D2 and D2.*Ppcd1* eyes in animals ranging from 2.5 to 3.5 months of age. ONL thickness for wildtype D2 animals ranged from 21 μm (adjacent to the optic nerve) to 67 μm, while ONL thickness of D2.*Ppcd1* animals ranged from undetectable to 48 μm. ONL loss was also present in D2.*Ppcd1 Gpnmb*^*+*^ animals (S5 Fig).

Focal areas of RPE cell hyperplasia adjacent to regions of photoreceptor atrophy are visible by light microscopy (Figs 3C and 4E). Cytoplasmic vacuoles are apparent. Ultrastructurally, cytoplasmic vacuolization, moderate accumulation of organelle debris and long cell membrane projections extending into the subretinal space (Fig 4F) are visible. Bruch’s membrane and the choroid appear normal.

## Discussion

Evidence of angle blockage and distension of the anterior chamber in D2.*Ppcd1* eyes is seen as early as postnatal day 5 [15]. The anterior chamber is clearly enlarged at postnatal day 8, suggesting that increased intraocular pressure develops at an early age in D2.*Ppcd1* animals. By 3 months of age, Tonolab measurements confirm that mouse PPCD1 produces a marked increase in IOP. This increase is of a larger magnitude and occurs at an earlier age than the IOP increase reported for wildtype D2 mice [24]. Although the corneal pathology seen in PPCD1 animals does decrease the sensitivity and accuracy of IOP measurements, the Tonolab measurements nevertheless demonstrate marked increases IOP in many D2.*Ppcd1* animals, which is consistent with gross observations of an enlarged globe and anterior chamber and a tendency of the globe to rupture upon enucleation. The large variation in IOP is puzzling. It is possible that IOP is initially increased in all D2.*Ppcd1* animals at an early age and that some compensatory mechanism, either developmental (corneal and globe enlargement or development of an alternate drainage pathway), or pathologic (ciliary body atrophy or phthisis) is acting to decrease IOP. This may be related to the observation that DBA/2J mice develop increased IOP beginning at 6 to 9 months of age as a result of iris atrophy and pigment dispersion, followed by decreases in IOP beginning after 12 months of age [25]. This late decrease in IOP has been attributed to ciliary body atrophy and decreased aqueous humor production [25, 26].

Consistent with the observed increase in IOP, D2.*Ppcd1* mice exhibit a decrease of approximately 20% in the number of cells in the RGCL, similar to that reported for retinal ganglion cell loss in an angle closure model of chronic IOP elevation [27]. A number of cell types are stained by the Nissl procedure and it is not possible to precisely determine the degree of retinal ganglion cell loss. However, a 20% decrease in axon counts was also observed, indicating the presence of optic nerve damage in D2.*Ppcd1* mice.

PPCD1 mice on the D2-*Gpnmb*^*+*^ background also develop corneal endothelial cell metaplasia and angle occlusion indistinguishable from that seen in D2.*Ppcd1* eyes. The increase in IOP also occurs at an earlier age than that reported for D2 mice, suggesting that the anterior chamber pathology of the PPCD1 mouse is independent of the *Gpnmb*^*R150X*^ mutation associated with iris disease and development of increased IOP and glaucoma in aged D2 mice. RGCL cell loss in D2.*Ppcd1* mice is also independent of the *Gpnmb*^*R150X*^ mutation.

In contrast to the relatively modest decreases in axon and RGCL cell numbers, D2.*Ppcd1* animals develop a severe degeneration of the outer nuclear layer in association with proliferation of the retinal pigment epithelium. Outer retinal pathology in PPCD1 eyes may be secondary to prolonged high intraocular pressure or inflammation or may be a direct effect of the PPCD1 mutation. Wang et al. have observed retinal detachment after an acute rise in intraocular pressure [28] and D2.*Ppcd1* animals exhibit evidence of retinal detachment. Proliferation and dedifferentiation of retinal pigment epithelial cells due to experimental retinal detachment [29, 30] have been reported to resolve over time; however, the continued high IOP present in D2.*Ppcd1* animals may prevent resolution of the RPE abnormalities.

Although IOP elevation has primarily been associated with inner retina cell loss in humans and in animal models [24, 25], there is increasing evidence for pathological changes in the outer retina. Nork et al described patchy photoreceptor loss in retinas from human glaucoma patients [31]. In the mouse, electroretinogram measurements have reported attenuation of scotopic a- and b-wave amplitude concurrent with thinning of the outer retina and the inner synaptic layer and decreases in ONL thickness in DBA and albino Swiss mice [27, 32–35]. ONL degeneration in D2.*Ppcd1* animals occurs substantially earlier and is more severe than that reported for D2 animals, is not dependent upon *Gpnmb*^*R150X*^, and is associated with proliferation of the retinal pigment epithelium.

Alternatively, RPE proliferation leading to photoreceptor death may be a direct effect of the PPCD1 mutation. *Zeb1*, the causative gene for human PPCD3, is expressed in the RPE, where it has been shown to play a role in initiation of proliferation, loss of pigment, and transformation to a fibroblastic state during RPE dedifferentiation. *Ovol2* is a transcriptional repressor of *Zeb1* [36] while *Csrp2bp* is a component of the histone acetyltransferase complex, ATAC, [37] and expressed in the RPE [16].

In conclusion, we have demonstrated that PPCD1 mice on the D2 background develop increased intraocular pressure and optic nerve damage, which is not dependent upon the *Gpnmb*^*R150X*^ mutation. Significant axon and retinal ganglion cell layer loss is observed by five months of age. By three months of age, D2.*Ppcd1* mice also exhibit outer nuclear layer degeneration and proliferation of the retinal pigment epithelium. Studies are underway to determine if RPE hyperplasia and ONL loss are direct or indirect effects of the PPCD1 mutation.

## Supporting information

S1 Fig D2.*Ppcd1* eyes at postnatal day 8. The arrows indicate the anterior chamber. c, cornea. Magnification is 40X and staining is H&E. A. D2 B. D2.*Ppcd1*.
(TIF)

S2 Fig D2.*Ppcd1* eye size. Photograph of enucleated eyes from D2.*Ppcd1* animals, age 3 months. (1) indicates an abnormally small PPCD1 eye, (2) a normal wildtype eye, and (3) an enlarged PPCD1 eye.
(TIF)

S3 Fig D2.*Ppcd1 Gpnmb*^*+*^iridocorneal angle. Arrow indicates epithelialized corneal endothelial cells occluding the iridocorneal angle. Age, 3 months. A. D2-*Gpnmb*^+^. B. D2.*Ppcd1 Gpnmb*^*+*^.
(TIF)

S4 Fig ONL loss in D2.*Ppcd1* retinas. H&E-stained retinal sections from 21 D2.*Ppcd1* animals, numbered 1 through 21, and one D2 animal (WT). Animals are 2.5 to 3.5 months old. Two eyes, A and B, were examined for animals 1 through 17 and WT. Only a single eye was available for animals 18 through 21.
(TIF)

S5 Fig D2.*Ppcd1 Gpnmb*^*+*^ retina. A. Retina of D2-*Gpnmb*^*+*^ animal, age 3 months. B. Retina of a D2.*Ppcd1 Gpnmb*^*+*^ animal, age 3 months.
(TIF)
