# Supplementary figures and images for: Retinal pathology in the PPCD1 mouse
Source: PLoS One. 2017 Oct 5;12(10):e0185094. doi: 10.1371/journal.pone.0185094 (PMC5628829; doi:10.1371/journal.pone.0185094)

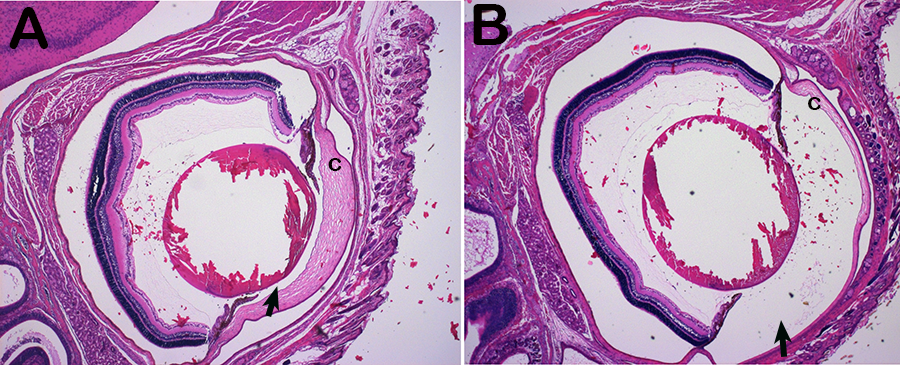

Supplement: S1 Fig — The arrows indicate the anterior chamber. c, cornea. Magnification is 40X and staining is H&E. A. D2 B. D2.Ppcd1. (TIF) [file pone.0185094.s001.tif]

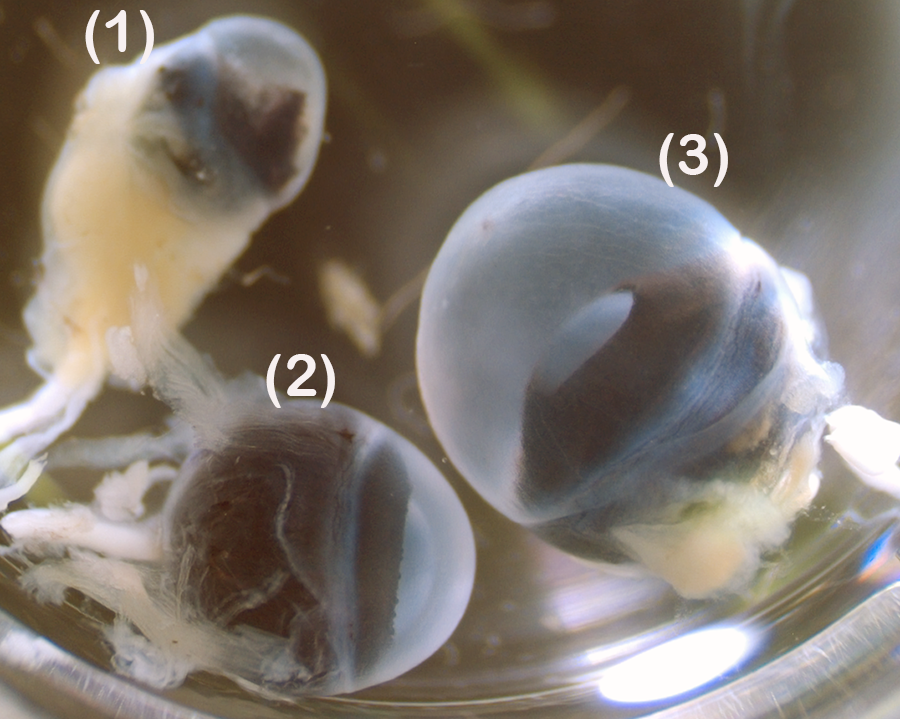

Supplement: S2 Fig — Photograph of enucleated eyes from D2.Ppcd1 animals, age 3 months. (1) indicates an abnormally small PPCD1 eye, (2) a normal wildtype eye, and (3) an enlarged PPCD1 eye. (TIF) [file pone.0185094.s002.tif]

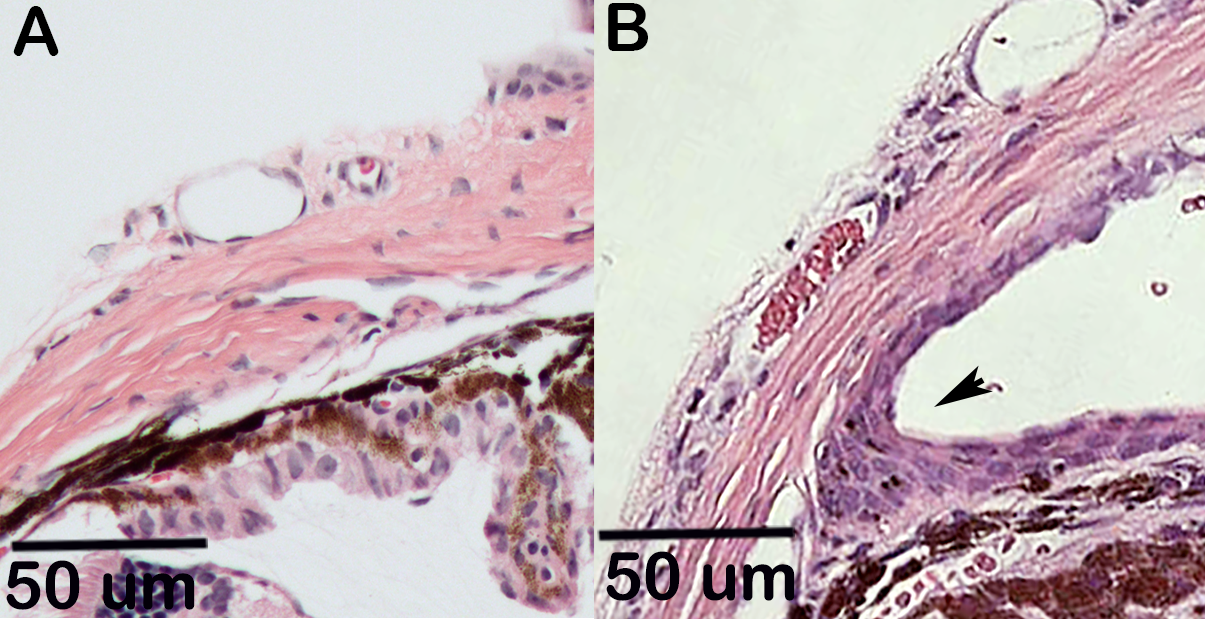

Supplement: S3 Fig — Arrow indicates epithelialized corneal endothelial cells occluding the iridocorneal angle. Age, 3 months. A. D2-Gpnmb+. B. D2.Ppcd1 Gpnmb+. (TIF) [file pone.0185094.s003.tif]

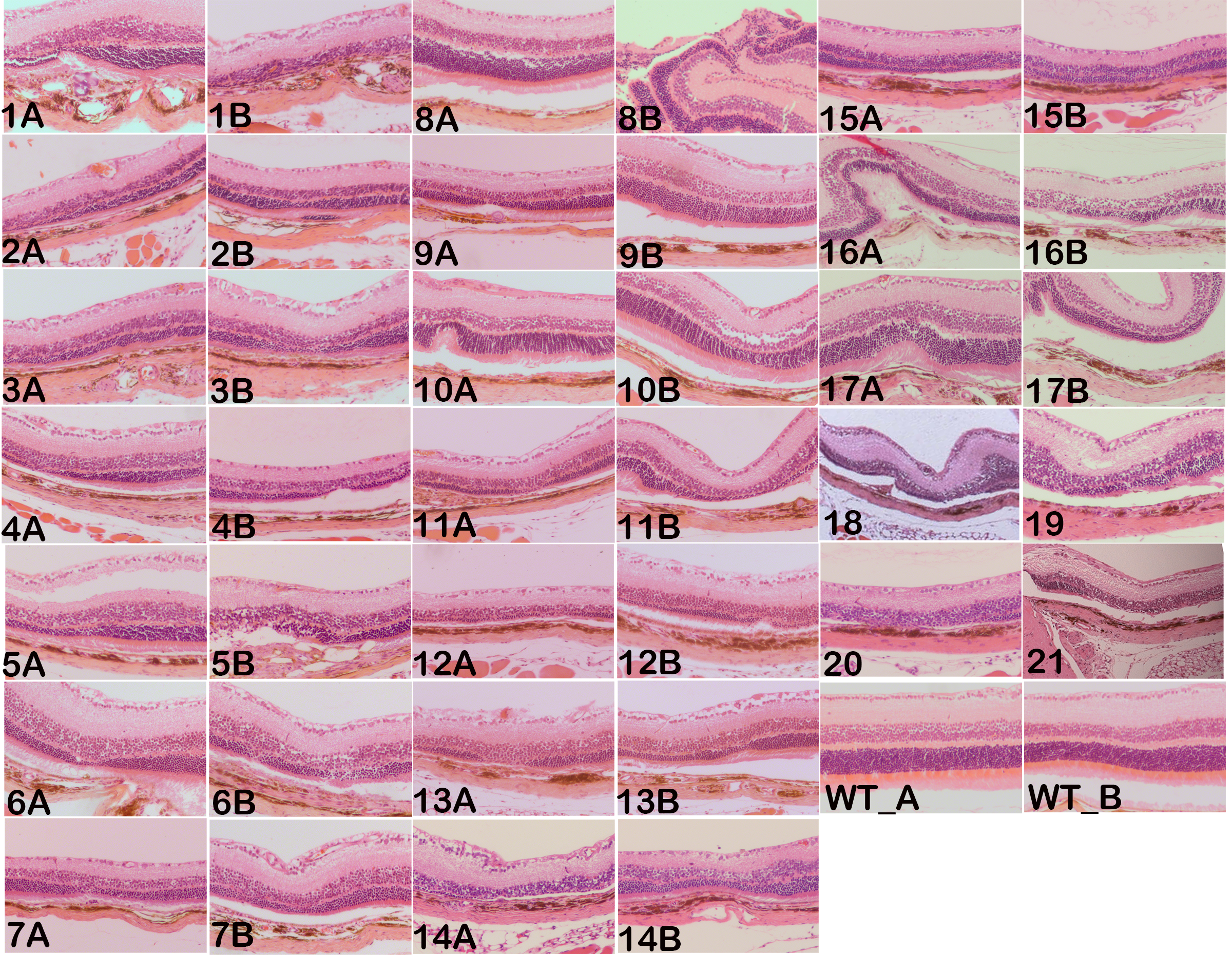

Supplement: S4 Fig — H&E-stained retinal sections from 21 D2.Ppcd1 animals, numbered 1 through 21, and one D2 animal (WT). Animals are 2.5 to 3.5 months old. Two eyes, A and B, were examined for animals 1 through 17 and WT. Only a single eye was available for animals 18 through 21. (TIF) [file pone.0185094.s004.tif]

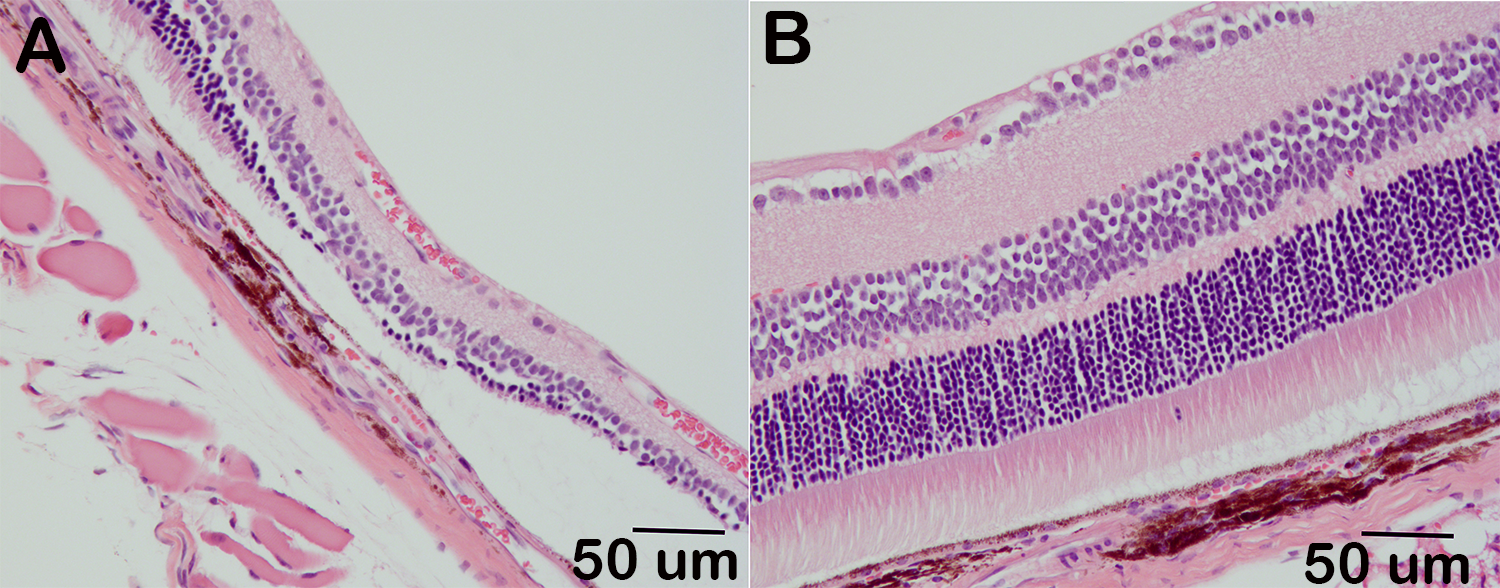

Supplement: S5 Fig — A. Retina of D2-Gpnmb+ animal, age 3 months. B. Retina of a D2.Ppcd1 Gpnmb+ animal, age 3 months. (TIF) [file pone.0185094.s005.tif]
